# ‘Pulling the world in and pushing it away’: participating bodies and the concept of coping

**DOI:** 10.1136/medhum-2018-011581

**Published:** 2019-07-09

**Authors:** Robbie Duschinsky, Samantha Reisz, Serena Messina

**Affiliations:** 1 Department of Public Health and Primary Care, University of Cambridge, Cambridge, UK; 2 Department of Psychology, University of Texas, Austin, Texas, USA; 3 University of Cambridge, School of Clinical Medicine, Cambridge, UK

**Keywords:** literature, medical humanities

## Abstract

In her lead article in this special issue, Monica Greco (2018) offers the concept of participating bodies as a ’possibility of conceiving bodies themselves—and bodily events such as disease/illness—as expressing values and perhaps even socially meaningful "preferences"’. Such a position seeks to avoid capitulation to a) an image of bodily processes as without values or responsiveness, object rather than participant; b) an image of human agents as unitary, self-knowing, sovereign choosers—unless ill. This article will explore this perspective as applied to the idea of coping. The article will explore strategies of everyday living, through particular consideration of Lauren Berlant’s reading of Two Girls, Fat and Thin by Mary Gaitskill. In her interpretation of the novel, Berlant assesses the kinds of problems for subjects and bodies that may be solved or managed through participation in or refraining from participation in thinking, food or sex. The account of coping and embodiment in Berlant’s reflections will then be placed in dialogue with findings by Alexandra Michel, who watched the process of physical burnout in investment banking associates during a 13-year cultural ethnography, observing as the bankers heeded or ignored the cues their bodies gave about the limits of feasible demands. The article as a whole offers an illustration of the value of Greco’s reflections for offering a fresh and valuable perspective on the concept of coping.

## Introduction

Bringing together two decades of writing and elaboration, Greco^1^ argues against two widespread positions: the idea of the multiplicity of bodily processes as without values or preferences, mere object rather than participant; and the idea of human agents as unitary, self-regulating choosers—unless ill. This, Greco suggests, is a false opposition. She proposes that we consider the possibility of bodies themselves, and bodily events such as illness, expressing values, aims and socially meaningful preferences. From this perspective, the concept of ‘psychosomatics’ takes on new colour. The term refers to more than the status of certain physical symptoms that are ‘medically unexplained’; it comes to refer to a domain of inquiry in which the anatomical, biophysical, social body is active as participant both in and beyond the production of symptoms. Such a perspective has special value in highlighting the zone between activity and passivity in the selection and stabilisation of our responses. Likewise, the domain of psychosomatics sketched by Greco, calls us to attend to the lag or mismatch between bodily participation and the social ‘self’, and how this discrepancy is implicated in the relation of behaviour and perception. Greco’s approach has roots in Canguilhem^2^ and Foucault,^3^ and is aligned with productive scholarship in a variety of domains, ranging from studies of physical coordination, pain and fatigue in sport,^4^ to critical disability studies,^5^ archaeological studies of ageing,^6^ phenomenological philosophy^7^ and feminist studies of endocrinology.^8–10^ Yet despite such strands of scholarship, in general there is still anxiety in the humanities about thinking bodily participation beyond the agentic subject, a residual tendency to think about embodiment as an effect.

This is especially true within discussions of biopolitics. Although there are clear exceptions,^11^ the body often appears as object or as a surface for inscription. One significant implication of Greco’s position is that it especially highlights the strategies through which human biological processes are managed in the context of wider relations of power and governance. Conventionally, biopolitics is framed as a concern with how i) biological processes, as object, are ii) oriented towards particular political goals iii) through modes of subjectivation, the production of certain kinds of desirable and undesirable ‘kinds’ of people.^12 13^ Greco’s position, however, would highlight the need to intensify developments in thinking about biopolitics that give attention to how vital processes exceed biopolitical capture. Vital processes may conflict with extant modes of governance in the expression of values and aims. These processes themselves may also, in Greco’s account, incorporate, resignify and exploit the norms implicit in biopolitical governance.^14^

For Greco, then, the bodily processes may themselves participate in biopolitics within the sphere of the everyday and its practices. In this, Greco does not require any concept of the body as a coherent, unitary entity; her interest is rather in exploring the multifaceted nature of bodily life and its contributions to the points of coherence and incoherence in human subjectivity, forms of valuation and social practices. This perspective is aligned with the work of the American studies scholar and queer theorist Lauren Berlant, particularly in her reading of the novel *Two Girls, Fat and Thin* by Mary Gaitskill.^15^ In a recent issue of *Medical Humanities*, Wasson^16^ has pointed to the fruitfulness of Berlant’s thinking for conceptualising ‘the grinding down of vulnerable subjects that occurs not through dramatic events but through structurally induced attrition’. In her interpretation of Gaitskill, Berlant assesses the kinds of embodied problems that may be solved or managed through participation in—or refraining from participation in—thinking, eating or sex under such conditions of attrition. These are three activities that at first sight appear wildly different. Considering them together would appear to be an ‘outrageous proposition’.^17^ Superficially, all that they seem to have in common is their structure of desire: the subject wishes to *have* the sexual object, the edible object, a thought. Although in such an account, the body serves merely as a unitary instrument or ancillary of the wish, to be disciplined in the achievement of the object of desire.

It is true that eating, thinking and sex are fundamental sites of self-governance. In this regard, bodily processes are an genre of object to be disciplined by the subject, to be marshalled in their potentialities to form effective human capital, amid unravelling arrangements of reciprocity and institutions. This is the familiar position of the body within many discussions of biopolitics. But, additionally, eating, thinking and sex are also embodied activities that can be deployed for making days that feel emotionally bearable, less poisonous for a time. For Berlant, these survival strategies can be conceived as negotiations *with* biopolitics, as ‘laboratories for an ongoing question of how life might be structured’ (p. 104).^18^ What marks eating, thinking, sex as a set for Berlant is that at times each can function as a part-active, part-passive strategy in which the intensities of bodies-in-environments are modulated into a felt state. All three activities hold out the prospect of making us feel less powerless, scattered and small in ourselves—even if in a sense their enactment contributes to our powerlessness, and we may know this. Bodily processes are irreducibly important participants here, if the goal of the practices is an *embodied affect*. In the latter part of the article, Berlant’s analysis will be placed into conversation with ethnographic observations over 13 years by Alexandra Michel of the process of physical burnout in investment banking associates. Whereas Berlant explores the participation of the body within strategies to make more bearable days, Michel documents the irruption of the body in ways that are so predictable that they form part of the expectable arc of labour within work-intensive, results-driven industries. Both, however, illuminate the body’s preferences and values that operate at the ground level of biopolitics. In this, Berlant and Michel offer helpful illustration, across quite different media and contexts, of the value of Greco’s perspective as applied to reflecting on what is entailed by coping.

## Eating, thinking and sex in *Two Girls, Fat and Thin*

As a first source for thinking about participating bodies within the conduct of coping, we will consider Berlant’s reading of three survival strategies within Gaitskill’s^15^ novel, and the role they play in offering access to an embodied feeling of resilience, if not necessarily the capacity to make better traction in the world. Although there is a wide literature debating the merits of the concept of resilience as a scholarly tool,^19 20^ Berlant’s analysis is unusual in attending to practices that have their goal the embodied feeling of some kind of ‘resilience’ (or, as Berlant sometimes puts it, ‘reprieve’). This terrain is almost always bypassed by discussions of ‘resistance’ in the social sciences and humanities, which retain an image of choice-making, severed from its conditions of possibility. In this way, the strategic aspects of embodied feeling remain too often out of focus, despite their contribution to the range of practices through which power and subjectivity are negotiated.^21^

The two protagonists of Gaitskill’s^15^ novel are Justine and Dorothy. Early in the novel, we learn that both live with memories of traumatic abuse and neglect from their childhoods. Justine was abused by a friend of her father’s when she was aged 5 years; Dorothy experienced repeated abuse from her father until she left home, beginning at age 14. The plot centres around an interview Justine conducts with Dorothy, who had served for a time as secretary to Anna Granite (a loosely fictionalised Ayn Rand). However, centre-stage in the book are the strategies the protagonists both use, despite their significant differences from one another, in responding to and coping with their experience of ‘a subtle, turbulent, cockeyed and exhausting world’ (p. 32).^15^ Each helps ‘organise life to lessen the impact’ (p. 112).^15^ For individuals well-ensconced within the conventional family form, eating, thinking and sex might well be integrated and tangled together in taken-for-granted, invisible routines of intimacy. As such, Berlant^22^ notes that one of the paradoxical possibilities of a novel is to dramatise the disaggregation of such practices. Specifically, she argues that the isolation experienced by Justine and Dorothy in *Two Girls, Fat and Thin* means that the strategies are articulated and distinguished, making their functioning clearer. Justine works for a doctor, Dorothy for a law firm (p. 77)^22^; observes that Gaitskill formulates their depictions as each a ‘case study’. The bleakness and extremity of their lives, as depicted by Gaitskill, helps to focus, highlight and burnish their practices of coping for the reader.

### Eating

The title of the book highlights food consumption as the form of coping most centrally in focus. Dorothy responds to repeated experiences of rape by her father in ways that hold tight to the goodness of food, against the threats of the world. Mentally, "I hugged the inner me to myself at night and thought how I had enjoyed dinner, no matter what" (p. 117).^15^ Physically, “I stopped brushing my teeth, except on rare occasions. All at once, I hated putting the paste-laden brush into my nice warm mouth" (p. 60).^15^ By contrast, Justine reduces her claims on the world, with a methodical reserve. She takes little pleasure from food, and her thin body participates in the performance of a pretty, ‘good girl’. Although consciously she knows that it is harmful for her health, Justine ‘could not make herself eat nutritionally sound food’ and ‘the eating of meals had somehow become burdensome to her’ (p. 278).^15^ When we do see Justine eat, ‘her stomach felt too tight even for chewed-up mouthfuls of salad, and she ate uncomfortably’ (p. 225).^15^ Berlant’s account of the food practices of Justine and Dorothy is complex, and has often been misunderstood by commentators.^23^ There are three common simplifications. A first is that Justine’s constriction of food and Dorothy’s appetite are merely physical qualities, without bearing for them as subjects. A second simplification is that these behaviours are conscious ‘coping strategies’, in the manner described by Lazarus and Folkman^24^ selected by Justine and Dorothy as purposeful, self-aware actors in response to their appraisal of the needs of the situation, such that they could easily talk about their actions and their causes. A third simplification is that the food practices are reducible to symptoms of childhood trauma, as expressions of mental illness. Part of the complexity of Berlant’s position is that she does not think that these accounts are simply wrong. It is true that there is a disjuncture or lag between weight and subjectivity, so one cannot be read off from the other. It is also true that the inhibition or consumption of food has a strategic quality in making more bearable days for the protagonists, and sometimes they know quite well what they are doing. And it is also true that the strategies evolved in, and initially responded to, the context of childhood suffering, although with time they have become elaborated and more than their childhood origins.

Yet Berlant^22^ argues that these three interpretations are facets of a deeper logic. For Gaitskill’s^15^ protagonists, food practices serve as a domain in which embodied processes are actively participating in the work of coping through modulating interchange between subject and world: ’for the girls, eating is a technique for pulling the world in and pushing it away according to their own terms and sense of pacing. It is neither an act of conscious intentional Agency, not a manifestation of unconscious symptoms’ (p. 86).^22^ It ’is a way of both being and not being in the world, giving the girls leverage to engage in exchange and to withdraw from sharing anything with just anyone’ (p. 83).^22^ Gaitskill is highly attentive to the meanings of eating practices, and of not eating, aware of our rich emotional investment in what we do not do. Whereas everyday life offers little pleasure or acknowledgement to either Justine or Dorothy, eating or not eating express and defend their desire to find a space in the world. Justine’s body participates in producing both the pride and the cool, relieving numbness evoked by self-denial and neglecting herself, with an affective quality somewhere between fear and achievement.^25 26^ Dorothy’s body participates in ‘creating a force field around her, seeming to neutralise what… might come from others—curiosity or attachment’ (p. 77).^22^ They pity one another for the price attached to the other’s use of food, and what it harms or inhibits. But each also admires what eating or not eating contains and organises for the other, physically and emotionally. Justine finds herself torn between contempt and admiration for Dorothy: ’The hell of it was, the fat woman was obviously very strong in some way. She had that craziness locked into formation, doing drills, getting her up and out and moving’ (p. 189).^15^

Berlant^22^ argues that part of our investment in Justine and Dorothy’s use of food is a sense of recognition, even if this usage may be more extreme and desperate than the reader’s. Berlant argues that, not just Gaitskill’s^15^ characters but for her readers too, ’food is one of the few spaces of controllable, reliable pleasure people have… At the same time that one builds a life the pressures of its reproduction can be exhausting. Eating can be seen as a form of ballast against wearing out, but also as a counter- dissipation, in that, like other small pleasures, it can produce an experience of self-abeyance, of floating sideways’ (pp. 115–116).^22^ In bodily participation in what and when to eat, the activity can provide precisely a kind of rest for the self, ’an interruption of being good, conscious and intentional that feels like a relief… and enable[s] people to feel more resilient in the everyday. This *sense of resilience* is different from actual resilience: bodies wear out from the pleasures that help them live on’ (pp. 26–27).^27^

In conceptualising the pleasures of eating or not eating, and how this helps bodies get through the day, Berlant invokes the concept of ‘self-medication’. The concept comes from theories of health behaviour, and is associated with the argument that food can be used as a form of self-soothing, especially when other forms of self-regulation such as social support are unavailable or would be difficult or emotionally costly to access.^28^ The concept of self-medication is invoked by behavioural scientists to interpret research findings linking trauma experiences to sustained overeating or undereating, in both clinical and community samples. For instance, Mason *et al* 29 asked a US-based community sample of participants about their mental health and their eating practices. The researchers found that experiences of finding oneself eating without wishing to were neatly associated, in a dose-response relationship, with the number of lifetime post-traumatic stress disorder (PTSD) symptoms. That two-thirds of the sample had at least one lifetime symptom of PTSD, suggested to the researchers that this is not a minority process associated with the most traumatised individuals, but a continuum on which most people are situated by degrees. Berlant^30^ agrees with the behavioural science literature that eating does seem to be a strategy that helps manage some kind of problem, and that there are both ‘usual’ and ‘catastrophic’ ends of the spectrum in the deployment of this strategy. However, whereas the behavioural science literature tends to imagine self-medication through eating as helping hold a damaged subject together (p. 115)^22^ offers an additional interpretation, seeing the pleasure and work of coping offered by eating and of not eating ‘as a kind of self-medication through self-interruption'.

Justine wants herself to eat; Dorothy is baffled that she finds herself eating. There is a lag or mismatch between the body’s evaluation of food and the subject’s. Berlant’s^22^ reading of the novel suggests that food practices offer some ease or break for Justine and Dorothy from the burdens of subjectivity, through the participation of bodily processes. This participation is shaped and made more intense by experiences of trauma and adversity, which through social, psychological and endocrinal process may draw the psychosomatic organism towards self-medication practices. Berlant is clear both i) that eating and not eating can be fuelled in part by forms of trauma, which can shape and alter the body’s values and preferences and ii) that this does not reduce eating in any simple way to an effect of the trauma. For Berlant, eating is an embodied practice, recruited and personalised to help organise and interrupt the social, economic and psychological problems that surface for the subject within their everyday life. Preferred responses are potentiated by social structures, resource availability, individual projects as well as by the repertoires and dispositions of the organism. For Berlant the very different relationships with food that Justine and Dorothy dramatise, in the aftermath of trauma, highlight that eating needs to be considered for what problems it helps solve or manage for a person-as-organism, to understand its locatedness and tenacity.

### Thinking

A second strategy identified by Berlant in *Two Girls, Fat and Thin* is thinking about the world, monitoring it. ‘Like eating’ (p. 87)^22^ observes, ‘monitoring appears to control the shape and pacing of exchange’. Thinking as an activity gets given a heavy emotional load for Justine and Dorothy, more than they seem to want or expect from it. Part of this load comes from the fact that, like our bodies, thinking promises not to withdraw or abandon us, since it *is* us—although naturally this promise may be betrayed. Across Gaitskill’s writings, this is a common theme. For instance, describing Joel in *Bad Behaviour* (p. 8)^31^ writes: "He could feel his eyes become clouded with privacy as he slipped discretely into a sheltering cave of sexual fantasy. His focus wobbled. He slipped out again". Turning attention to thoughts, whether private fantasy or surveillance of the world or absorption in a book, is treated by Gaitskill as making a recess within the streaming of experience, within which that streaming can eddy; it allows internal space to be a destination, rather than in the moment feeling the need for objects and destinations from the environments.^32^ This permits intense investment in guarded thoughts without expression or with minimal expression. Just like eating or not eating, thinking—or, at times, concertedly not thinking—are practices that enable for Justine and Dorothy the embodied affect of affectionate contempt for the world, a great buffer for difficult, unpredictable and exhausting days.

As Savransky^33^ has observed, our familiar frames of reference for conceptualising thinking are as the expression of the sovereign, rational self or as something we passively have and undergo. Both frames of reference more or less approximate our experience at times. But neither adequately accounts for the contexts and conditions of thinking. In particular, everyday life repeatedly insists to each of us that bodily processes participate in the process of thinking, an observation that has long been basic for cultural theory and to the medical humanities^34 35^ and to work on embodied reading within literacy studies.^36 37^ There are physical states in which thinking is enlivened or deadened. An implication of Greco’s position on psychosomatic subjects is that endocrinal and nutritive processes shape and are shaped by the intensity, colour and direction of our thoughts. Attention to the affects of psychosomatic strategies such as eating and thinking point to the participation of non-representational, underspecified but nonetheless meaningful and evaluative responses by the body to the environment. Berlant makes an acute point well aligned with Greco’s perspective when she urges us to ’think about the way energy drinks, sugar, and caffeine fuel the process of simply getting by. Think of the imperative to focus amidst distraction, and the imperative to stay in a decent mood. Working the workday requires fatigue management, efficiency and affective labour at work and later at home’ (p. 28).^27^ Certainly, the body that contributes to the experience and effect of stimulants is always in part a social construction, depending on their context, branding and other forms of discursive location. However, the properties of the body, and its resourcing or depletion, make a contribution to this construction—according to both theory^2 38–40^ and the results of randomised placebo-controlled research in psychopharmacology.^41 42^

Greco and Savransky^43^ draw an important distinction between three concepts that are usually elided: care of the self, technology of the self and practice of freedom. Foucault and  Rabinow p. 225)^44^ define technologies of the self as ’techniques by which individuals effect … a certain number of operations on their own bodies and souls, thoughts, conduct, and way of being, so as to transform themselves in order to attain a certain state of happiness, purity, wisdom, perfection, or immortality". Eating and thinking practices are often recruited as technologies of the self. But in Gaitskill,^15^ they do not serve that function. They are not strategies for developing the self, although development may or may not be a byproduct. They are, however, profound practices of the care of the self, as forms of coping. And the freedom that they offer is not in the first instance an escape from modes of domination, but freedom from the relentlessness of the tiredness and powerlessness of being oneself. Justine and Dorothy are not seeking to make themselves better through their eating and thinking practices, but to feel each day as more bearable. In Greco’s^38^ terms, biological individuals are never entirely isolated, and yet an organism is identifiable as such by its partial capacity to maintain its own difference and modes of normativity. In short, the body irreducibly participates in this process in pulling the world in and pushing it away, as eating and thinking or their inhibition support the attempt to feel resilient in the moment, sometimes against the social ambitions and desires of the subject herself.

### Sex

Finally, a third strategy identified by Berlant in *Two Girls, Fat and Thin* is the use of sex. This strategy operates somewhat differently to the other two in the novel. Eating and thinking open up ‘an infinite domain… directed towards an enigmatic somewhere. In contrast, what the girls value most about sex is its unoriginality’ (p. 94).^22^ For Justine and Dorothy sex is so saturated by conventions that it offers the prospect of the feeling of embodied resilience precisely by providing a chance to be invisible within the body. In our society, Berlant argues ’conventionality in the penumbra of sex provides relief from the ordinary muddles that arise in the intimate zones of encounter with other persons and the world’ (p. 6).^18^ We offer others only what we expect they can stand, and in doing so we make space for bodily processes to participate as embodied intensities organise all that is desirable and concerning about intimacy. Berlant argues that, within these parameters, sex is little about achieving fulfilment from the object, and much more a space of relationality in which hopes, expectations and anxieties about intimacy are negotiated to make them less unbearable. Yes, sex in the novel is destabilising for Justine and Dorothy. But, in such cases, it may still be part of the work of coping: ’what counts as composure might be a conventional style of instability rather than an instability that actually threatens the subject’s core patterning’ (p. 93).^22^

Berlant observes that sex with love is such a powerful symbol in our culture, and specifically such a powerful symbol of simplicity, that this makes the use of sex to achieve other purposes, such as reprieve or detachment, relatively unacknowledged. She criticises accounts of sexuality built around "some kind of hydraulics of the drive, which I’m not at all sure is correct about the subject’s rise and fall of intensities. For example, I could want to want to have sex, but not feel like it, but put myself in the position to have it in order to generate the energy to spread out a little, to dismantle, without there being that sense of internal pressure implied in discharge".^45^ Mistaking sex as in the first instance as about desire, as the available terms of femininity encourage Justine and Dorothy to do, makes it difficult for them to acknowledge the normative cruelties and exploitation associated with the conventions that saturate sex for them. It makes them wonder a little why they are doing it, why they experience their bodies as pulling towards sex that they regret or would prefer not to repeat.

Indeed, Dorothy mainly finds sex a confusing, somewhat physically distant experience. Justine finds her body simultaneously pulling towards the closeness of sex and flinching away from the pain of sex, when as a subject she is feeling neither attraction nor repulsion but rather numb, worn and a bit desolate. Yet ’she recognised something compelling in it, a ghastly compulsion akin to that of a starving lab animal which will keep pressing the button that once supplied it with food, even though the button now jolts its poor small body with increasing doses of electric shock’ (p. 227).^15^ Attempting to understand the lag or mismatch between body and subject, Berlant argues that conventionalised modalities and their attendant emotional and/or physical pain are not beside the point for both Justine and Dorothy. They have intense investment in low-intensity sex. For the protagonists this kind of sex opens up a space of reciprocity through embodied unoriginality and, therefore, reprieve. However, like eating and thinking, Gaitskill is attentive to the moments when sex as part of the work of coping starts to fail in this function. Justine and Dorothy’s experiences of reassuringly unoriginal sex offers modulation of exposure to the world. Nonetheless, sex risks leaving them more face to face with others than either intended or quite wanted, which is how the novel ends. Gaitskill’s^15^ ending emphasises that the strategies adopted by her protagonists are no more than ways to cope, since to do more than cope would require resources, support and forms of control that they do not have. Yet, by the end, the book leaves open whether the operation of the three forms of coping has brought Justine and Dorothy together as ‘an attachment that might become a solidarity that could produce more and better traction in the world’ for each of them, some path towards thriving (p. 161).^22^

Looked at through Greco’s account of the psychosomatic subject, it is notable that in eating, thinking and sex the relationship between subject, physicality and reality is made salient: the materiality and ephemerality of food; the subjective reality and material unreality of thoughts; sex as the truth of one’s being, or not. And in making this relationship salient, and inserting it against the other demands of a day, the self is permitted some feeling of reprieve, of not having been overturned by the world. Each is a strategy that is at once localised and with implications for the whole electrical grid of felt experience, no matter how difficult things might otherwise be. It is this that makes eating, thinking and sex both the basis for a sense of ordinariness, and something that punctuates the ordinary. This is suggested by Berlant’s attention to the ‘consumption of food and the production of intense intellection’ which reveals that ’each, like sex, is a process of absorption and a way of being in the world, a way of bringing it in, entering it, and averting it. While optimistic, these habituated modes of being are also techniques of self-annihilation and negation, ways of using the episodic pain of particular exchanges in order not, for a minute, to be that ordinary failed person, with that history’ (p. 81).^22^

In each strategy, practices are engaged in which the will becomes bendy or sleepy, out of focus and the body’s values, aims and preferences participate in producing the feeling of a less bad day. As Dorothy puts it, rather heartcatchingly: "I had enjoyed dinner, no matter what" (p. 117).^15^ The participation of bodily processes is highlighted by the fact that all three strategies have as their goal, not the achievement of the object, but an embodied feeling. They are absolutely dependent on bodily participation, in a mode of activity that is sometimes conscious, sometimes unconscious but often neither strictly the expression or enactment of cognitive will nor involuntary and merely encountered. Notably, this account is aligned with the perspective that has increasingly been taken by the wider literature of coping research since Lazarus,^46^ in contrast to earlier work which presumed that coping strategies must be conscious expressions of will. In part, this recognition in the coping literature has come from integration with work in developmental psychology, and the developmentalists’ insights into the origins of tacit or nonconscious processes. Likewise, examining at Gaitskill’s narration of the lives of Justine and Dorothy, Berlant is continually concerned to emphasise how these strategies are built developmentally, from childhood: for the protagonists, over time, each strategy comes to be ‘wound about her so completely she no longer knew it was there’ (p. 153).^15^ Most of the time the work of coping inhabits the region of life that is not surprising when it comes to attention but which ‘falls under the radar when someone asks you how your day was’.^47^

## Bodily attrition

Shilling (p. 74)^48^ has observed that ‘in rejecting the negative aspects of naturalistic views’, scholars have ‘tended to neglect how the body forms a basis for, and contributes towards, social life’, although even he is not inclined to ascribe as much participation to bodily processes as does Greco. True, unconscious dynamics have been highlighted by psychoanalytic approaches, and not-fully-conscious processes have been considered by phenomenology and affect theory. Yet in both cases, unconscious and affective processes are distinguished but then often reabsorbed by the subject, with bodily processes just along for the ride rather than as participant. This seems in part motivated by knee-jerk worries about sociobiology. The idea that biological processes could have values or preferences is seen as a naturalistic fallacy, and a dangerous essentialism. And of course fallacious and essentialising treatments of biology do have such consequences, especially where a static and antiquated image of biology is invoked and where the body’s authority is invoked in various ways in making a particular message have the force of moral inevitability. However, this is by no means the only way of considering the psychosomatic.^38^ In the forms of coping under discussion here, the preferences of bodily processes are difficult to ignore, as an absurd amount of life is spent coaxing or inhibiting propensities to feel sleepy, empty, sharp, tossed about or exhilarated under certain conditions. As Greco’s work highlights, this activity of coaxing and inhibiting is important for the way that biological processes construe and interrupt modes of subjectivation, and hence the relationship between the subject and modes of governance and self-governance.

For instance, the capacity of firms and universities to focus their attention on minds and social relationships occurs on the basis of assumptions that subjects will take care of their own bodies within the everyday, keeping them from intruding on overcommitment and overwork, and focusing on mind and interpersonal successes when narrating the self. Greco^1^ cites Whitehead’s remark that, although mind/body dualism is reductionist and often unhelpful, we should not fail to do full justice to the positive achievements enabled by this outlook. There are unquestionably major advantages to environments that value people for their perspectives. Yet, at another level, an institutional environment that disavows care for or valuation of the consequences for the overcommitted and overworked organism is a recipe for burnout. In fact, exhaustion and illness form part of the expected trajectory and selection process for workers in results-focused sectors like investment banking, academia and social work.^49 50^ Michel conducted a 13-year ethnography of two cohorts of Wall Street banking associates that effectively illustrates these processes, observing ’dualism in the wild’ (p. 67).^51^ She criticises certain tendencies in theorists such as Marx and Foucault who sometimes focus too readily on how the body is disciplined by power, as a passive object. She reports that the body changed in its role as participant over time, such that descriptions of institutional logics of power orchestrating docile bodies could stand as relevant descriptions of entry into the workplace, but not of what happened over time.

In their first 3 years of work in the sector, Michel observes, bankers were intent on disciplining bodily processes and on denying or relativising the participation of bodies in their work:

When I asked bankers about physical needs such as sleep, they made stark distinctions between the body and the mind, giving priority to the mind, like the following Bank A vice president: “I totally believe in mind over matter. There are no such things as physical needs. Tell me one physical need and I can tell you a culture in which they have controlled it" (p. 54).^51^

They were supported in this bifurcating process by tactics deployed by their firms:

"Our most important currency is not time but energy. It is easy to keep people at work around the clock. Minds are willing. You have to fight the biology" (Bank В director). Senior bankers explained how the constant buzz of open floors' layout facilitated long hours because it impeded reflection and nervously stimulated bankers. The banks also offered food and caffeine at strategic times, namely, "when people’s blood sugar slumps and that gives you energy to keep going" (Bank A associate) (p. 319).^52^

Yet by the fourth year, bankers started to mention the physical and biological needs in interviews with Michel without prompting. The interviews depicted the body as strangling subjects from the inside, choking their capacity to work or as taking revenge (p. 342).^52^ Michel, who had worked as an investment banker before entering academia, started to feel some of these familiar sensations as she tried to keep up with her participants’ 100-hour work weeks as an ethnographer. She saw the rhythms of investment banking impacting her blood pressure, emotional responses and sentence length when writing.^53^ From her longitudinal ethnographic work with her participants, she reports that some kind of physical breakdown was universal among the bankers in her sample between their fourth and sixth year in the sector:

Starting in year 4, the bankers’ bodies started to break down under overwork, causing bankers’ self-narratives to change. The body now became a frequent theme. Its role changed from that of an unproblematic object to being a hostile antagonist. Between years 4 and 6 all bankers had proceeded to this stage, which I refer to as "body-as-antagonist". Committed to their work, bankers responded to the body’s rebellion by pushing harder to maintain performance. Starting in year 6, for about 40 percent of bankers, the breakdowns were so severe that they had to give up fighting their bodies (p. 53).^51^

Some bankers stopped fighting low energy and heeded it as a cue, and came to recognise the physical fallout of inactivity and prolonged sitting (and, paradoxically, for some the fallout of treating exercise like investment banking, and pursuing it without pity or respite). Michel^52^ observes that in some cases this change of attitude contributed to creative work strategies and greater compassion to colleagues that, in fact, benefited the banks, although Michel observes that their colleagues mocked these participants as weak. Some bankers facing breakdown entered cycles, oscillating between rest and kicking their bodies into action, working yet longer hours to compensate and treating symptoms like back pain and insomnia as just background noise. And some bankers left the sector, although Michel was surprised that the transition changed little for these workers in their relationship with their bodies, as they kept to some version of their old routines. From the sixth year onwards, Michel (p. 348)^52^ noted a change in the banker’s use of the ‘I’ pronoun: whereas before they implicitly meant their mind when they said ‘I’, experiences of physical breakdown caused disruptions that distanced themselves from and objectified a mind unable to sufficiently control the production of symptoms. They described entering into negotiation with their bodies: "I see my body is a friend who has always supported me as best as she could, ignored her. I always thought so highly of my mind, but it has let much more often than my body" (Vice President of Bank B) (p. 350).^52^

Michel’s account is of one kind of high-strain workplace. Public health researchers have also documented the predictable health consequences of other kinds of high-strain working environments, such as precarious employment and perceived job insecurity.^54^ Workplace demands can contribute to embodied responses, most notably discomfort and pain, that interrupt the subject’s efforts as contractor of their labour. As Michel observes, these might be understood as mere noise, without integral relevance to the subject. However, they can also be understood as cues that the organism is approaching certain limits.^55^

Michel’s discussions of the role of participating bodies in the thresholds of sustainability for the workplace chimes with the biopolitics of everyday life displayed in the three strategies—eating, thinking and sex—highlighted by Berlant’s analysis. In each case, Berlant examines the psychosomatic domain of coping and surviving, in which bodies express values, aims and socially meaningful preferences. The same can be said of whether and how Justine and Dorothy dress, drink tea, hug themselves for comfort, regulate their breathing to stay calm, attempt to sleep, travel around the city, arrange things around their apartments—other psychosomatic survival strategies in *Two Girls, Fat and Thin*,^15^ although these are not discussed explicitly by Berlant. In each, bodily participation and preferences in engagement, estrangement and exchange with the world serve as a technique through which exposure to experience is modulated through the participation of the body in the interruption of the subject. They contribute as part of the ground-level processes of biopolitical governance, but without necessarily producing a coherent subject-position in the manner that many discussions of biopolitical governance suppose.

## Conclusion

For the protagonists of Gaitskill’s *Two Girls, Fat and Thin,* 15 and Berlant^18 22^ argues for its readers, strategies such as eating, thinking and sex contribute to a feeling of resilience, interrupting the terms of insertion into the day. Whereas for Gaitskill’s protagonists, bodily processes participate in the characters’ modulation of intensities and interruption of self-sovereignty to achieve the feeling of resilience, among Michel’s research participants, the psychosomatic body signals limits to what can be demanded of the organism by results-oriented work. In the latter case, a variety of bodily processes interrupt the bankers’ attempts to maintain discipline and self-sovereignty. Some recognise these as cues regarding the limits beyond which further demands will be costly, others just attempt to push on through. As such, a contrast lies in the fact that Berlant emphasises the participation of the body in the work of coping through the production of felt resilience, whereas Michel documents ways that bodily processes may participate in signalling approach to what is bearable or sustainability. Yet both analyses have in common that they highlight the participation of the multiplicity of bodily processes and the organism’s modulation of intensities in the domain of everyday biopolitics. Bodily processes are active in stimulating and shaping responses to constraints and environments, and their ensuring pain and pleasure, aims and shames, discipline and leisure. This participation does not entail a naturalistic fallacy in which ‘the body must be listened to’, echoing the trope of the wise body pulling towards how things should be. The eating practices of Justine and Dorothy are hardly ones that will contribute to their long-term health; the symptoms of Michel’s bankers signal the potential for burnout, but do not in themselves possess normative force. Both Gaitskill’s characters and Michel’s participants enter and exit awareness, depending on what else is going on for them, that there is a trade-off between their long-term physical health and the needs of another equilibrium which is experienced as more pressing. Justine is in fact pulled in both directions by her body in relation to sex: wanting harmful sex and wanting also to escape. The participation of bodily processes, even in the work of coping, depend on what values are assigned to health and to a life.
